# Development and Validation of a Histological Method to Measure Microvessel Density in Whole-Slide Images of Cancer Tissue

**DOI:** 10.1371/journal.pone.0161496

**Published:** 2016-09-01

**Authors:** Koen M. Marien, Valerie Croons, Yannick Waumans, Ellen Sluydts, Stefanie De Schepper, Luc Andries, Wim Waelput, Erik Fransen, Peter B. Vermeulen, Mark M. Kockx, Guido R. Y. De Meyer

**Affiliations:** 1 Division of Physiopharmacology, University of Antwerp, Antwerp, Belgium; 2 HistoGeneX NV, Antwerp, Belgium; 3 Department of Pathology, University Hospital Brussels (UZ Brussel), Brussels, Belgium; 4 StatUa Center for Statistics, University of Antwerp, Antwerp, Belgium; 5 CORE (Translational Cancer Research Unit, GZA Hospitals), Faculty of Medicine and Health Sciences, University of Antwerp, Antwerp, Belgium; Universita degli Studi di Bari Aldo Moro, ITALY

## Abstract

Despite all efforts made to develop predictive biomarkers for antiangiogenic therapies, no unambiguous markers have been identified so far. This is due to among others the lack of standardized tests. This study presents an improved microvessel density quantification method in tumor tissue based on stereological principles and using whole-slide images. Vessels in tissue sections of different cancer types were stained for CD31 by an automated and validated immunohistochemical staining method. The stained slides were digitized with a digital slide scanner. Systematic, uniform, random sampling of the regions of interest on the whole-slide images was performed semi-automatically with the previously published applications AutoTag and AutoSnap. Subsequently, an unbiased counting grid was combined with the images generated with these scripts. Up to six independent observers counted microvessels in up to four cancer types: colorectal carcinoma, glioblastoma multiforme, ovarian carcinoma and renal cell carcinoma. At first, inter-observer variability was found to be unacceptable. However, after a series of consensus training sessions and interim statistical analysis, counting rules were modified and inter-observer concordance improved considerably. Every CD31-positive object was counted, with exclusion of suspected CD31-positive monocytes, macrophages and tumor cells. Furthermore, if interconnected, stained objects were considered a single vessel. Ten regions of interest were sufficient for accurate microvessel density measurements. Intra-observer and inter-observer variability were low (intraclass correlation coefficient > 0.7) if the observers were adequately trained.

## Introduction

Tumor growth can only be achieved when sufficient blood vessels are present in the tissue. Two major processes can be responsible for the blood supply: sprouting angiogenesis or, alternatively, co-option of existing blood vessels of the host [1]. Angiogenesis is triggered by vascular endothelial growth factor (VEGFA), which is produced in the tumor [2]. The molecular mechanisms of vascular co-option, on the other hand, have not been fully elucidated yet. The most frequently used technique to quantify the result of angiogenesis or vascular co-option in a tumor section is based on measuring the microvessel density, i.e. counting the microvessels at a high magnification (200x—400x) in a predefined number of fields [3–6]. In several cancer types, microvessel density has been quantified (e.g. in colorectal carcinoma (CRC), glioblastoma multiforme (GBM), ovarian carcinoma (OC) and renal cell carcinoma (RCC) (Table 1)) and found to be prognostic for survival [7]. However, not the same unit of measurement was used (e.g. microvessels per mm^2^ and microvessels per 200x field), data were highly variable and therefore difficult to compare, as illustrated by mean microvessel density in CRC ranging from 6 to 351 across several studies. In order to visualize microvessels, tumor sections have been stained immunohistochemically for one or multiple pan-endothelial markers, such as CD31 [8–10], CD34 [10–12], von Willebrand factor [10,12,13], endoglin [10], and/or coagulation factor VIII [10]. Hitherto, microvessel counting has mostly been performed in a fraction of the total tissue area determined by a sampling method such as the vascular hotspot method (Weidner’s method). This method involves the selection of one to five areas with the highest density of microvessels (hotspots) at low magnification, and the counting of vessels in these areas at high magnification [14,15] by computerized image analysis systems [16,17] or by applying a Chalkley grid [18]. In a previous study, we used a systematic uniform random sampling (SURS) method to avoid observer-dependent sampling variation and selected a limited number of at least five regions of interest (ROIs) on each whole-slide image (WSI) [19]. In these ROIs two parameters were measured by using an unbiased array of test points (grid) separated by constant distances: the number of vessel profiles per area (Q_A_ or microvessel density) and the number of grid points overlapping with vessels per area (A_A_) [20]. This sampling and counting method had previously been compared with other schemes, such as the hotspot method [21]. At the intra-observer level, the methods have variations of the same magnitude (coefficient of variation (CV) around 20%) [21]. At the inter-observer level, the SURS estimate of Q_A_ from the whole tumor section and the Chalkley method had the lowest variation (CV around 21%) with a small contribution by observers (CV 8% to 9%) [21]. Although the SURS estimate appeared the most reliable method to pick up microvessel density differences between study subjects [21,22], a major drawback is that it is labor- and time-intensive, limiting its use [21]. SURS has only been extensively studied in breast cancer [10,21,23], without applying digital tools to automate the process and without a thorough validation that is necessary for clinical use [5,24]. Accordingly, we developed and validated a method to measure microvessel density by using computer-assisted manual SURS of WSIs of cancer tissue, named AutoTag and AutoSnap, which reduces workload and guarantees full traceability [19]. In the present study, we investigated the intra- and inter-observer variability of this method in CD31-stained tissue sections of four different cancer types and in samples that have different spatial distributions of blood vessels.

**Table 1:** Results of different vessel counting methods in four cancer types. Studies differ in staining of the vessels, number of ROIs, magnification, number of observers and study size.

Cancer	Method	Stain	ROIs	Magn	Obs	mean ± SD MVD	med MVD	n	Ref
CRC	C	CD34	4	200	2	NA	4	30	[49]
CRC	C	CD34	3	200	1–2	NA	8	235	[50]
CRC	R	CD31	10	200	1	71 ± 22	NA	242	[51]
CRC	R	CD31	5	320	1	18 ± 12	15	106	[52]
CRC	W	CD31	3	200	2	114 ± 56	NA	210	[7]
CRC	W	CD31	3	200	1–2	115 ± 39	NA	178	[53]
CRC	W	VWF	5	200	2	21 ± 12	NA	132	[54]
CRC	W	CD34	3	200	1	76	NA	114	[55]
CRC	W	CD31	5	320	1	70 ± 40	59	106	[52]
CRC	W	CD31	3	400	2	19 ± 8	NA	87	[56]
CRC	W	CD34	3	400	1	32 ± 15	28	60	[57]
CRC	W	CD34	3	200	1–2	NA	28–33	56	[58]
CRC	W	CD31	5	100	1	35 ± 4	30	40	[59]
CRC	W	CD31	5	200	1	NA	75	116	[60]
CRC	W+CIAS	CD105	2	200	1	6± 5	5	15	[61]
CRC	W+CIAS	CD31	3–4	400	2	351 ± 40	NA	4	[16]
GBM	C	CD34	5	200	1	5	NA	62	[18]
GBM	R	CD34	5	200	1	NA	56	62	[18]
GBM	W	CD34	1	200	3	NA	77	97	[62]
GBM	W	CD34	1	200	1	NA	67	233	[62]
GBM	W	CD34	1	200	3	NA	84	114	[63]
GBM	W	VWF	3	200	2	33 ± 36	NA	55	[64]
GBM	W	CD34	3	200	1	84	NA	54	[65]
GBM	W	CD105	5	200	2	NA	31	40	[66]
GBM	W	collagen IV	1	250	1	200	NA	20	[67]
GBM	W	VWF	3	400	1	21 ± 12	NA	16	[68]
GBM	W	CD31	10	100	2	>100	NA	12	[69]
RCC	R	CD31/34	10	200	2–4	53	NA	70	[70]
RCC	W	CD31	2	250	1	NA	10	208	[9]
RCC	W	CD34	3	200	1	102 ± 23	NA	128	[71]
RCC	W	F8	1	400	1	741 ± 394	NA	97	[72]
RCC	W	CD34	5	400	2	98 ± 63	NA	70	[73]
RCC	W	CD31	8	400	1	NA	25	62	[74]
RCC	W	CD34	5	200	2	49 ± 20	NA	46	[75]
RCC	W	CD34	5	200	2	142	NA	36	[76]
RCC	W+CIAS	CD34	5	200	1	NA	124	87	[77]
RCC	W+CIAS	CD31	5	200	1	278 ± 62	NA	18	[17]
OC	NA	CD31	NA	NA	NA	14 ± 7	12	94	[78]
OC	R+CIAS	VWF	max	NA	1	NA	39	235	[79]
OC	W	CD31	3	200	1	21	NA	190	[80]
OC	W	CD34	4	200	1	NA	21	40	[81]
OC	W	CD34	6	200	2	NA	12	113	[82]
OC	W	CD34	5	200	1	62 ± 22	NA	62	[83]
OC	W	CD34	5	400	2	5 ± 1	NA	91	[84]
OC	W	CD34	3	200	2	NA	30	213	[85]
OC	W	CD31	3	400	1	NA	14	41	[86]
OC	W+CIAS	CD31	4	NA	1	35 ± 13	NA	46	[87]
OC	W+CIAS	VWF	max	NA	1	NA	88	235	[79]

CRC, colorectal cancer; GBM, glioblastoma multiforme; OC, ovarian cancer; RCC, renal cell cancer; C, Chalkley; CIAS, computer image analysis system; R, Random; W, Weidner; CD, cluster of differentiation; F8, coagulation factor VIII; VWF, von Willebrand factor; max, maximum possible; NA, not available; Magn, magnification used; Obs, number of observers; SD, standard deviation; MVD, microvessel density; med, median; Ref, reference number

## Materials and Methods

### Materials

**Fig 1:**
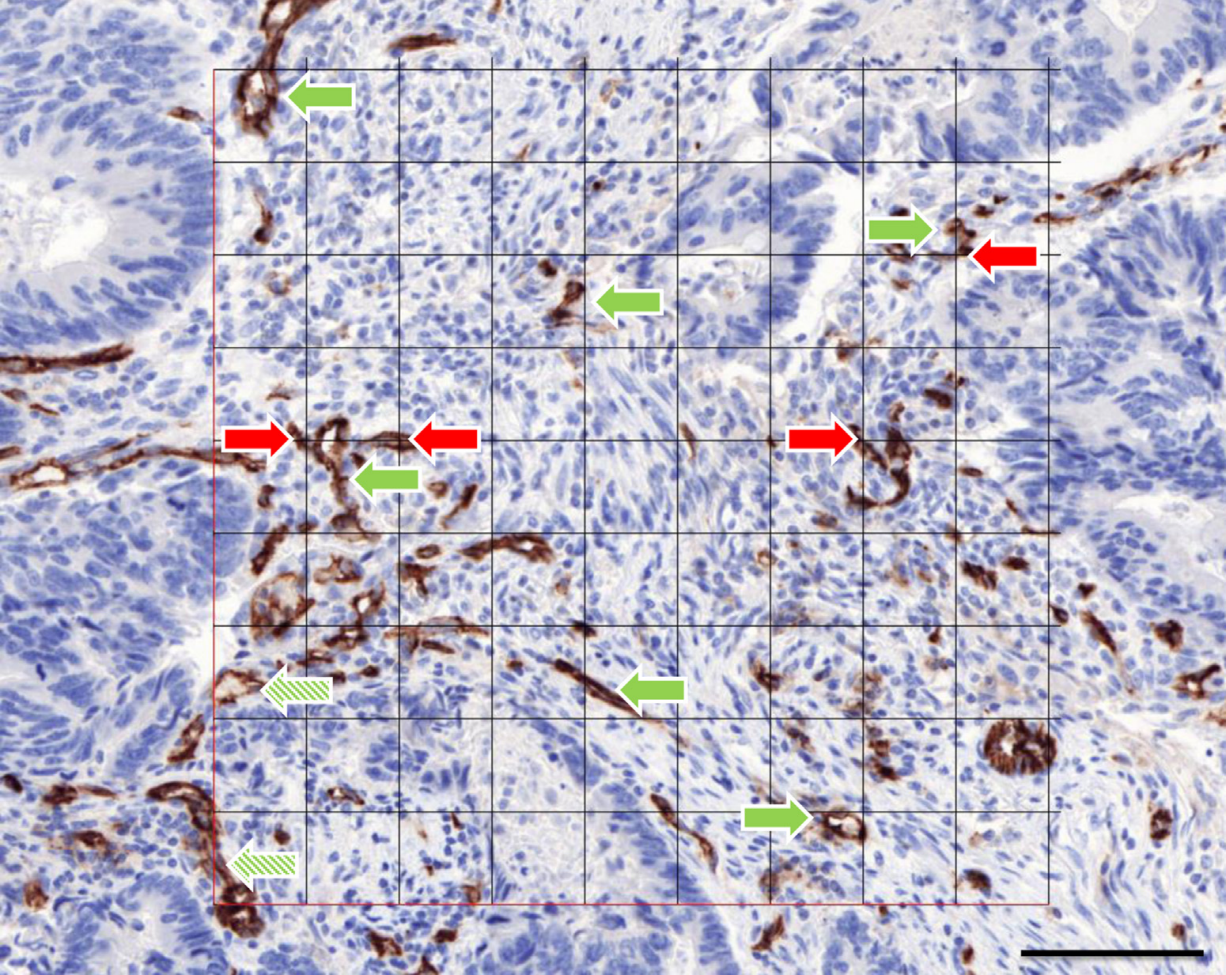
Example of a region of interest. It was captured in Pannoramic Viewer (3DHISTECH, Budapest, Hungary) and combined with a digital 81-points grid in Adobe Photoshop CS4. CD31-stained vessel profiles in the grid were counted as N (green arrow). Vessel profiles that cross the virtually extended left or lower line of the grid were not counted (shaded green arrow). The grid points that hit a CD31-stained vascular profile were counted as V (red arrow). Scale bar represents 100 μm.

WSIs were made of existing sections stained for CD31 from our database for inclusion in this study. Samples were coded to protect the privacy of patients. All samples were obtained in accordance with the Helsinki Declaration of 1964 and the study was approved by the local ethics committee (Ethisch Comité UZA/UA). For this type of study formal consent is not required. The sections were already stained using the NCL-CD31-1A10 antibody (Leica Biosystems, Diegem, Belgium) on a Benchmark^®^ XT platform (Ventana, Basel, Schweiz). The WSIs were created with a Pannoramic SCAN digital slide scanner (3DHISTECH, Budapest, Hungary) using a Zeiss plan-apochromatic objective (magnification: 20x, numerical aperture: 0.8) and a Hitachi (HV-F22CL) 3CCD progressive scan color camera (resolution: 0.2325 μm/pixel). JPEG image encoding with quality factor 80 and an interpolated focus distance of 15 with stitching in the scan options were chosen. For every slide a specific scan profile was configured and holes in the scan area were filled to allow for correct detection of tissue and in-focus images of the tissue. Scanned images were examined in Pannoramic Viewer (3DHISTECH) to check for image quality and to confirm that the whole tissue section was captured. The immunohistochemistry staining and imaging were carried out at HistoGeneX NV (Antwerp, Belgium), a Clinical Laboratory Improvement Amendments-certified laboratory that is accredited by CAP and the Belgian Accreditation Organization (ISO 15189). Four types of cancers were selected: CRC (19 patients), GBM (20 patients), OC (21 patients), and RCC (22 patients). Only samples with an area that could fit more than nine assessable ROIs were considered for selection. Finally, a set of samples was selected based on a representation of both low and high microvessel density heterogeneity in the study group. Two observers (KM and VC) trained and experienced in counting microvessels performed initial measurements. Four extra observers (ES, PV, WW, YW), two of which did not have previous experience in vessel counting (YW, WW) and two pathologists (PV, WW), were trained (30 minutes) and performed follow-up measurements on the same set of samples to assess inter-observer variability. SURS of 15 ROIs in the WSIs was done twice with Pannoramic Viewer (3DHISTECH, Budapest, Hungary) by one observer (KM) using a 20x magnification (Fig 1) assisted by the AutoTag and AutoSnap applications [19]. The second group of ROIs did not overlap with the ROIs from the first group. The first group of ROIs was used for assessing the intra- and inter-observer variability, while the second group was used for the inter-ROI variability. Guided by a pathology report of the closest hematoxylin and eosin-stained section, regions were taken in viable tumor tissue according to the SURS principle, but regions with abundant necrosis, inflammation, or ulceration were discarded. All images were analyzed on identical, color-calibrated displays. To assess intra-observer variability, vessels were counted at two different time points (with an interval period of one month) to allow washout of the visual memory [25]. A web-based viewer (Pathomation BVBA, Antwerp, Belgium) was used to guarantee traceability when analyzing the grid-combined images of the ROIs. Pathomation software allows combining data forms and WSI in the same viewport assuring that measurement results and the sample IDs stay unequivocally linked.

### Stereological point counting

All grid points overlapping with vessels (V) were counted, regardless of whether the microvessels crossed the left or bottom outer grid lines (Fig 1). A grid point, which was designated by two perpendicular cross-lines, was regarded as overlapping a microvessel when it fell on an endothelial cell or a vessel lumen (red arrows versus shaded red arrow in Fig 1). When, exceptionally, only a single endothelial cell of a larger vessel was stained, all other endothelial cells that lined this vessel were nonetheless counted upon intersection. To establish a reference area, all grid points intersecting with tissue (V_ref_) were counted. Small necrotic zones within tumor structures or glandular lumens were considered as cancer tissue. Only if more than 75% of the grid area (more than 60 out of the 81 grid points) covered tissue, the ROI was analyzed. The unbiased estimation of the microvessel areal fraction was calculated for each sample according to: $A_{A}\;=\;\frac{\sum_{i}V_{i}}{\sum_{i}V_{i,ref}},\;$ with an i value from 1 to 15 ROIs, expressed as a percentage of microvessels per area, with V_i_ the number of grid points overlapping with vessels in ROI i and V_i,ref_ the number of grid points hitting tissue in ROI i [19].

### Microvessel counting

Besides the stereological point counting, the microvessel density (Q_A_) is captured by our method. The outer borders of the superimposed grid (Fig 1) [19] delineated the counting chamber [20]. Vascular structures crossing the virtually extended left or bottom lines of the grid were not counted. Regardless of staining, the others were counted (shaded green arrows in Fig 1) [20]. The initial counting rules only took into account stained structures with a clear lumen or without a lumen but larger than one tumor cell. Accordingly, very small cross-sectioned capillaries without a clear lumen were not counted. CD31 staining of suspected myofibroblast-like cells or of cells not belonging to a blood vessel was also excluded for counting. Because of high inter-observer variability using these counting rules, the following, adapted counting rules were defined in which every CD31-positive object, no matter how small, should be counted, except suspected CD31-positive monocytes, macrophages and tumor cells. Furthermore, if CD31-positive objects were connected, they were considered a single object, while absence of staining defined two or more separate objects. microvessel density was calculated for each sample according to: $Q_{A}\;=\;\frac{\sum_{i}N_{i}}{\sum_{i}V_{i,ref}}$_,_ with an i value from 1 to 15 ROIs, expressed as number of microvessels per area, with N_i_ the number of counted vessels in ROI i and V_i,ref_ the number of grid points hitting tissue in ROI i [19].

### Statistical analysis

Heterogeneity of microvessel distribution was determined for every cancer type by calculating the difference between the minimum and maximum number of counted microvessels per ROI (N) in one sample. Heterogeneity was considered low or high when this difference was respectively below or above the median of the calculated differences for all the samples in the database of that cancer type. The average of two repeated measurements for each of the two observers (KM, VC) was used for the calculation of inter-observer variability. A script was written in the statistical package R (version 3.2) to perform the calculations and plotting [26]. The intraclass correlation coefficient (ICC) was calculated by using the icc(ratings, …) function from the irr package. A two-way model and type agreement was chosen. The unit of analysis for N and V was ‘unit’, whereas for Q_A_ and A_A_ it was ‘average’. The Kruskal-Wallis Rank Sum Test was carried out with the kruskal.test(formula, data, …) function from the stats package. XY plots of the counts with prediction intervals, Bland-Altman plots and Tukey boxplots were also constructed. Two-way ANOVA and paired Student t-test were performed in R. The minimum number of ROIs required for analysis of microvessel density was calculated using random sampling with replacement, also known as bootstrapping [19].

## Results

### Spatial Distribution of the Blood Vessels

**Fig 2:**
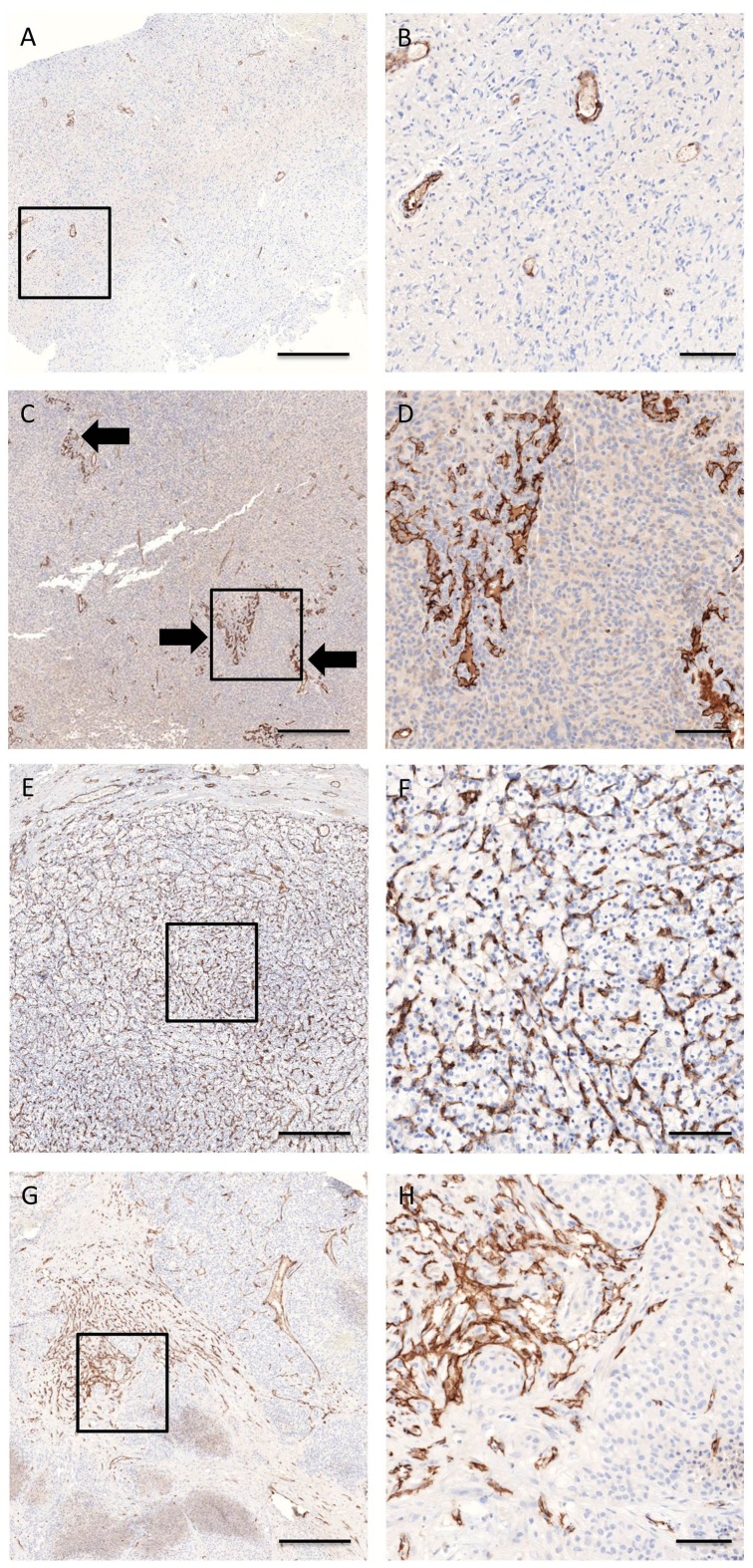
Histological heterogeneity of CD31-stained blood vessels in glioblastoma multiforme (a-d) and renal cell carcinoma (e-h). (a-b) Q_A_ = 15 vessels per mm^2^, A_A_ = 1.56%, (c-d) Q_A_ = 77 vessels per mm^2^, A_A_ = 3.70%, (e-f) Q_A_ = 183 vessels per mm^2^, A_A_ = 13.10%, (g-h) Q_A_ = 81 vessels per mm^2^, A_A_ = 6.17%. Low (a, b, e, f) heterogeneous samples showed a uniform distribution of vessel profiles as compared to high (c, d, g, h) heterogeneous samples. In glioblastoma multiforme, hotspots and garlands (arrows) were more easily recognized in heterogeneous than in homogeneous samples. Scale bar represents 500 μm (a, c, e, g) or 100 μm (b, d, f, h)

The mean microvessel density (Q_A_) and its standard deviation for the four cancer types was: 112 ± 50 (RCC), 76 ± 21 (CRC), 65 ± 30 (GBM), and 43 ± 13 (OC) vessels per mm^2^. The mean areal fraction of the vessels (A_A_) and its standard deviation for the four cancer types was: 11.92% ± 6.74% (RCC), 3.87% ± 1.18% (CRC), 4.70% ± 2.38% (GBM), and 2.99% ± 1.84% (OC). The median heterogeneity and its standard deviation for the four cancer types was: 31 ± 20 (RCC), 21 ± 8 (CRC), 22 ± 11 (GBM), 21 ± 6 (OC). The calculated heterogeneities of the spatial distribution of the microvessels were also visible in the images of the tumor tissue sections from the two groups (Fig 2). For example, in GBM, hotspots and/or ‘garlands’ could be more readily recognized in strongly heterogeneous samples compared to weakly heterogeneous samples (Fig 2A–2D). In RCC, highly vascularized regions can be uniformly distributed or are heterogeneously present across the tumor area (Fig 2E–2H).

### Inter-ROI variability

Comparing Q_A_ and A_A_ for two groups of non-overlapping ROIs (n = 15) in the same sample (n = 6) revealed that the choice of locations of the ROIs only affected A_A_. The calculated ICCs for Q_A_ were always above or equal to 0.8 (CRC: 0.9, GBM: 1.0, OC: 1.0, and RCC: 0.8), whereas the ICCs for A_A_ were significantly lower (CRC: 0.6, GBM: 0.6, OC: 0.7, and RCC: 0.7) (p = 0.01; paired Student t-test). The point-counting (A_A_) was sensitive to the choice of locations of the ROIs, whereas the profile-counting (Q_A_) was more robust with regard to the choice of locations.

### Minimum number of ROIs for accurate microvessel density measurements

**Fig 3:**
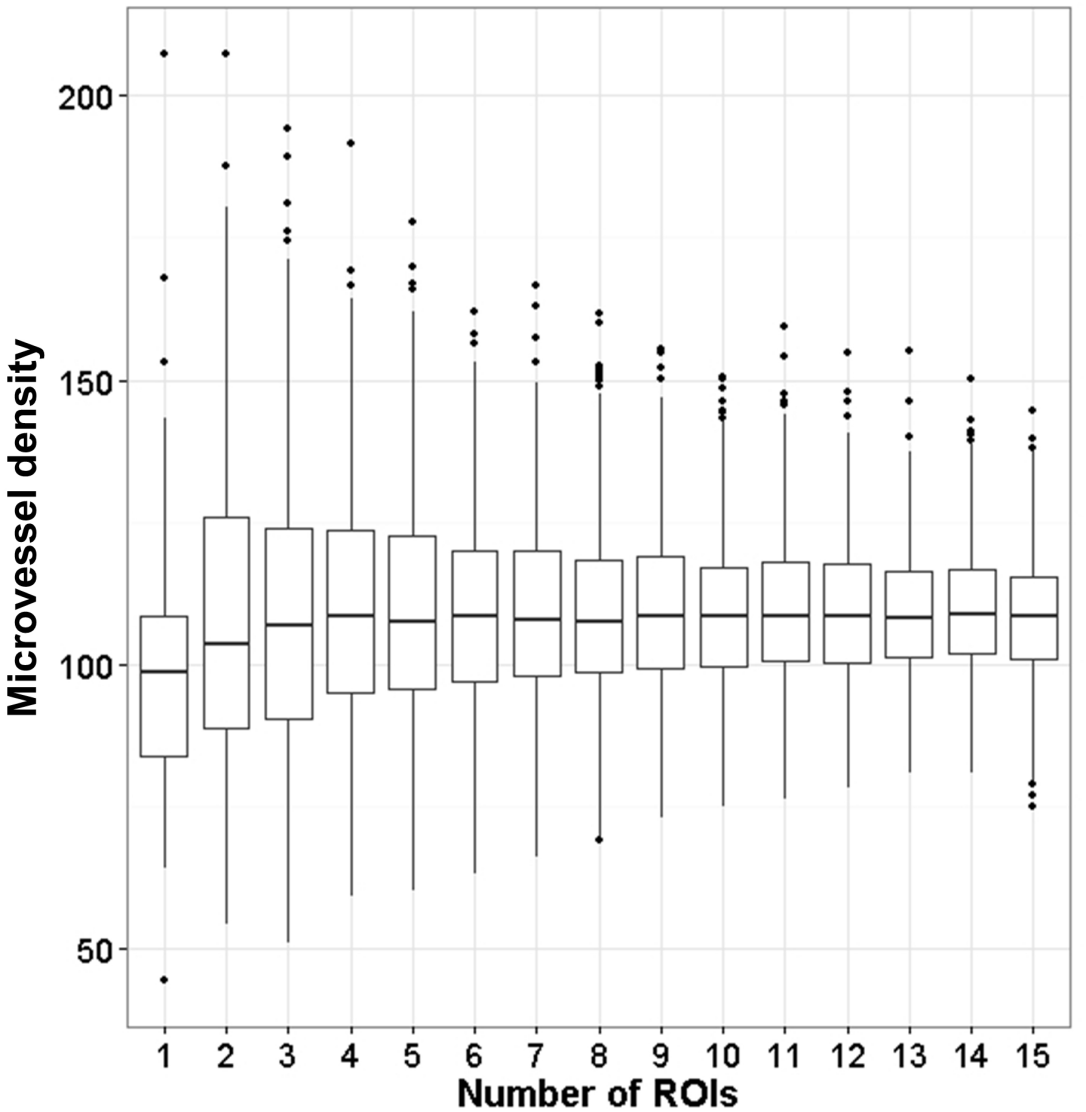
Distribution of 1000 bootstrap results. Here the results for CRC sample 19 is displayed. These were calculated based on the counting by the second observer during the second round of counting. Tukey boxplots were constructed for amounts of regions of interest evaluated. Ten regions are sufficient for accurate microvessel density calculation.

**Fig 4:**
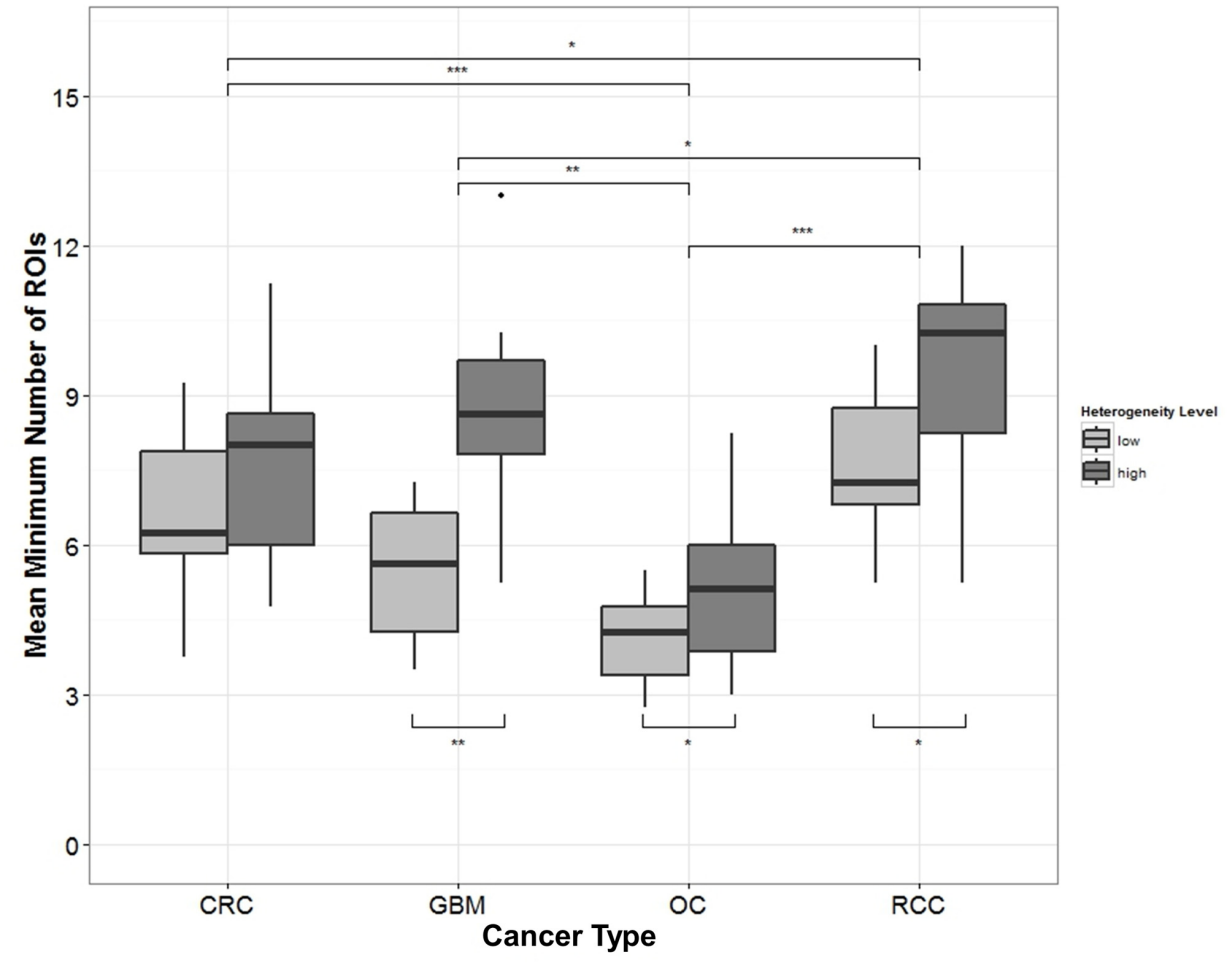
Tukey boxplots illustrating the relationship between the mean minimum number of regions of interest (ROIs) and the topological blood vessel heterogeneity. This was done for each sample and for every cancer type: colorectal carcinoma (CRC), glioblastoma multiforme (GBM), ovarian carcinoma (OC), and renal cell carcinoma (RCC). If the topological blood vessel heterogeneity of the samples increased (low < high), the minimum number of ROIs on average increased as well. ***p < 0.001, **p < 0.01, *p < 0.05.

A plot from a bootstrap analysis showed higher variation at lower number of ROIs compared to higher number of ROIs (Fig 3) [19]. Creating these graphs for 19 colorectal carcinomas, 22 renal cell carcinomas, 21 glioblastomas, and 21 ovarian carcinomas, counting ten ROIs appear to be sufficient for accurate microvessel density measurements [19]. Highly heterogeneous samples require more ROIs compared to samples with low heterogeneity (Fig 4). A relationship with heterogeneity was established by two-way ANOVA (CRC: p < 0.05; GBM: p < 0.001; OC: p < 0.01; RCC: p < 0.001). Moreover, there was a relationship with the observer (CRC: p < 0.001; GBM: p < 0.05; OC: p > 0.1; RCC: p < 0.05), implying that the required number of ROIs can differ between observers. On average, the minimum number of ROIs required was 5 for OCs, 7 for CRC and GBM, and 9 for RCC.

### Intra-observer variability

**Fig 5:**
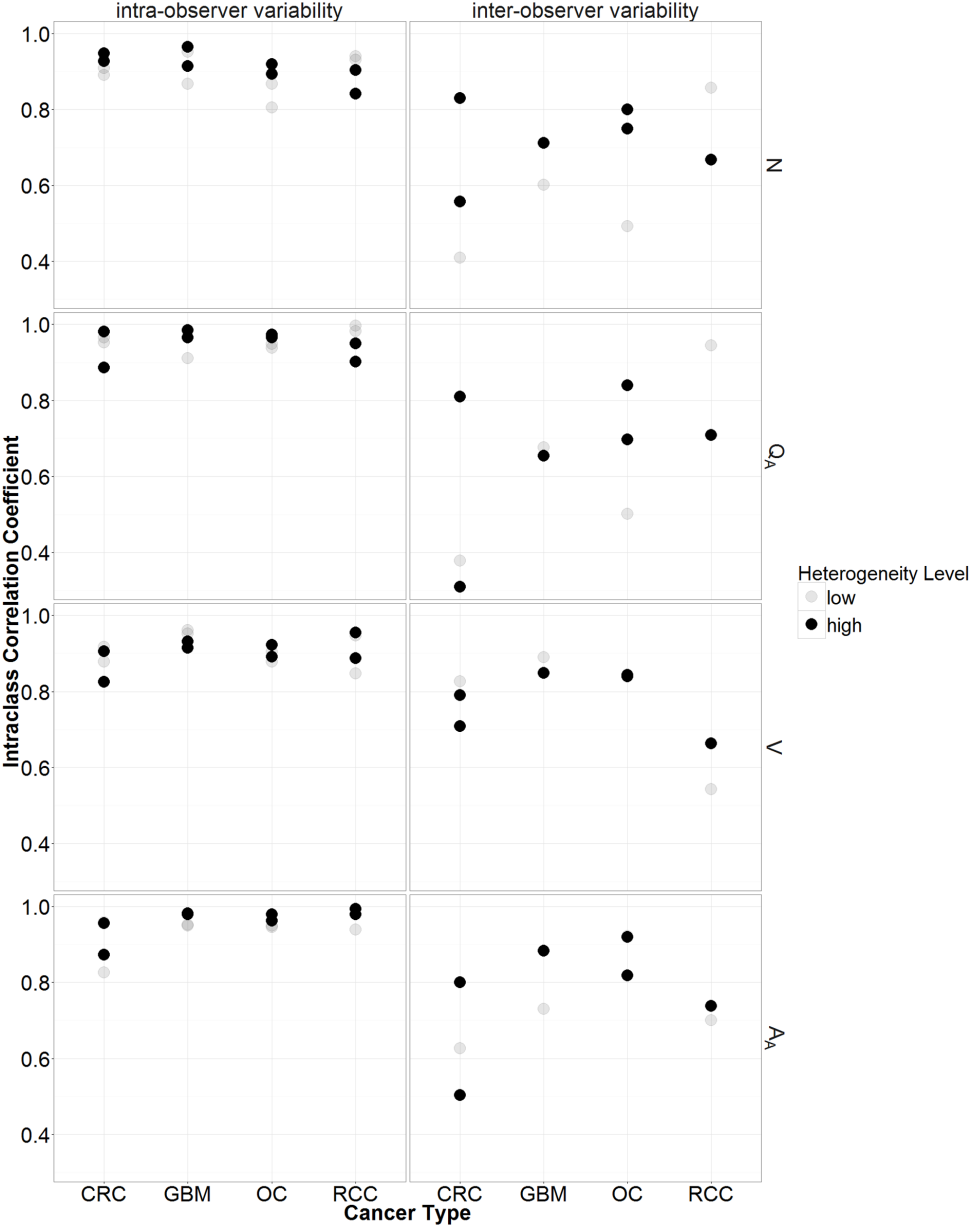
Intra- (left) and inter-observer (right) variability for the old counting rules. This was calculated by the intraclass correlation coefficients (ICC) for the four parameters (V, N, Q_A_, A_A_). In the first row this is displayed for the number of vessel profiles in a region of interest (N). In the second row this is displayed for the microvessel density (Q_A_). In the third row this is displayed for the number of points in the grid hitting a vessel profile in a region of interest (V). In the last row this is displayed for the areal fraction of vessel profiles (A_A_). In addition are the ICCs in relation to the heterogeneity level (low or high) and the cancer type (colorectal carcinoma (CRC), glioblastoma multiforme (GBM), ovarian carcinoma (OC), and renal cell carcinoma (RCC)) shown.

All ICC-values for the four parameters (N and Q_A_, V and A_A_), in the four cancer types and for both observers (KM, VC) were higher than 0.7 (Table 2), which is generally considered the minimal acceptable reliability [27]. Importantly, 81% of ICC-values were higher than 0.9, which is considered excellent concordance [27]. The ICC-values for CRC were lowest, those for GBM highest. The parameters Q_A_ and A_A_ showed lower intra-observer variability compared to N and V (Fig 5).

**Table 2:** Intra-observer variability for the old counting rules. This was calculated by the intraclass correlation coefficients (ICC) between the counting of round one and two of observers 1 and 2 (ICC1 and ICC2) for the four different cancer types and the four different parameters.

Cancer	Parameter	Samples	ICC1	ICC2
**CRC**	V	285	0.85 (0.78–0.89)	0.91 (0.86–0.94)
**CRC**	N	285	0.93 (0.89–0.95)	0.94 (0.92–0.96)
**CRC**	Q_A_	19	0.92 (0.79–0.97)	0.98 (0.95–0.99)
**CRC**	A_A_	19	0.87 (0.66–0.93)	0.96 (0.88–0.98)
**GBM**	V	300	0.94 (0.92–0.95)	0.93 (0.90–0.94)
**GBM**	N	300	0.92 (0.90–0.94)	0.97 (0.96–0.97)
**GBM**	Q_A_	20	0.98 (0.91–0.99)	0.99 (0.97–1.00)
**GBM**	A_A_	20	0.98 (0.95–0.99)	0.98 (0.92–0.99)
**OC**	V	315	0.90 (0.84–0.93)	0.92 (0.88–0.95)
**OC**	N	315	0.89 (0.84–0.92)	0.91 (0.87–0.94)
**OC**	Q_A_	21	0.97 (0.94–0.99)	0.96 (0.91–0.98)
**OC**	A_A_	21	0.97 (0.93–0.98)	0.98 (0.88–0.99)
**RCC**	V	330	0.88 (0.84–0.92)	0.95 (0.94–0.97)
**RCC**	N	330	0.87 (0.84–0.91)	0.92 (0.90–0.94)
**RCC**	Q_A_	22	0.95 (0.89–0.97)	0.96 (0.91–0.98)
**RCC**	A_A_	22	0.97 (0.94–0.99)	0.99 (0.99–1.00)

CRC, colorectal cancer; GBM, glioblastoma multiforme; OC, ovarian cancer; RCC, renal cell cancer; V, the number of grid points overlapping with vessels in one region of interest (ROI); N, the number of microvessel profiles in one ROI; Q_A_, the microvessel density based on N in one sample (15 ROIs); A_A_, the microvessel areal fraction based on V in one sample (15 ROIs); (LoCI—UpCI), the 95% lower and upper confidence intervals

### Inter-observer variability

**Fig 6:**
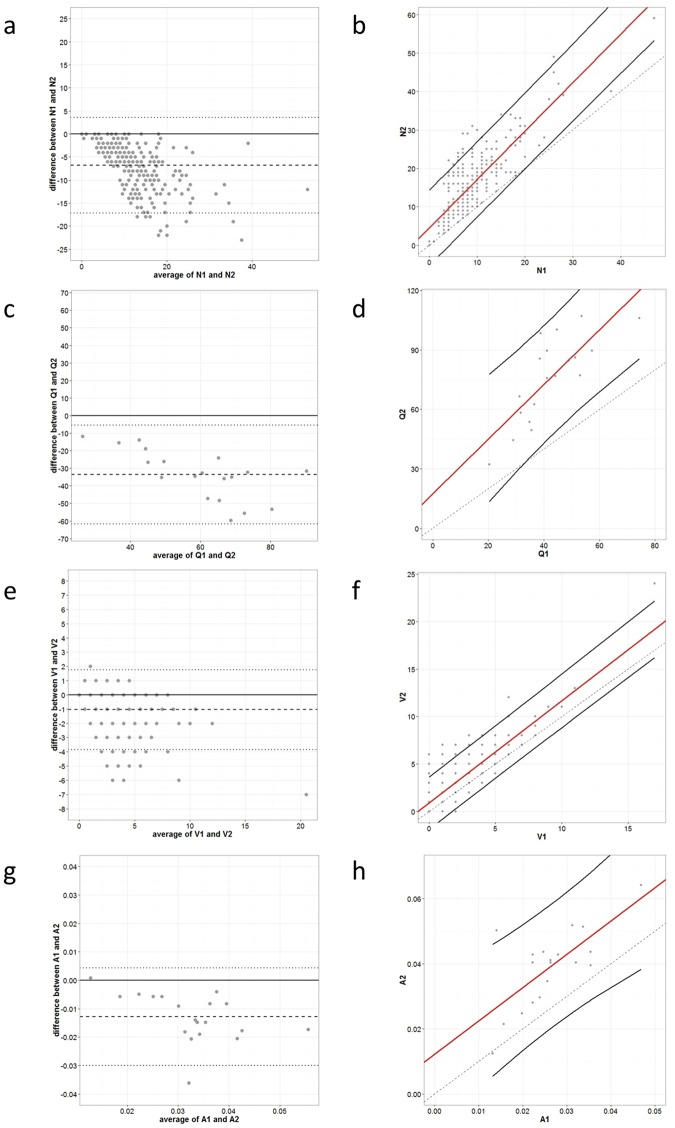
Inter-observer variation for the old counting rules between observer 1 (KM) and 2 (VC) for colorectal cancer samples. This was displayed by Bland-Altman (a, c, e, g) and prediction plots with prediction intervals (two black lines) (b, d, f, h) for the number of vessel profiles (N) (a, b), the microvessel density (Q_A_) (c,d), the number of points in the grid hitting a vessel profile (V) (e, f) and the areal fraction of vessel profiles (A_A_) (g, h). A systemic bias for N, Q_A_, and A_A_ was present as illustrated by the prediction plots (large distance between the x = y line (black and dashed) and the linear regression line of the measurements (red)).

Inter-observer variability ICC-values for the four parameters (N and Q_A_, V and A_A_) did not exceed 0.7 in all four cancer types (Fig 5, Table 3). The variability of N, Q_A_, and A_A_ was large in the CRC samples (Fig 6), which might be due to a large systematic bias between the observers. Therefore, a third trained and experienced observer (ES) quantified the samples. The ICC-values for the variability between observer two and three were better (0.9, 0.8, 0.9, and 0.8 for respectively N, Q_A_, V, and A_A_), but this was not the case between observer one and two, and observer one and three. Therefore, a series of consensus training sessions (two hours in total) was held in which the most discrepant cases were discussed. Accordingly, the following new counting rules were proposed: every CD31-positive object, no matter how small, should be counted, except suspected CD31-positive monocytes, macrophages and tumor cells. Identification of these cell types is not straightforward. For example, very dense inflammation prohibits accurate counting. Furthermore, if CD31-positive objects were connected, they were considered a single object, while absence of staining defined two or more separate objects. Using these new counting rules, three different observers (KM, PV, YW) recounted the CRC samples with the strongly discrepant inter-observer counts when the initial counting rules were used and the OC samples. A fourth observer (WW) counted only the OC samples. Both pathologists (PV, WW) recommended the exclusion of one sample from each set (OC1 and CRC7) as there were too many CD31-positive inflammatory cells. The inter-observer variability ICC-values resulting from the new counting rules were all greater than 0.7. Importantly, more than half of ICC-values exceeded 0.8 (Table 4). The parameters Q_A_ and A_A_ showed lower inter-observer variability compared to N and V.

**Table 3:** Inter-observer variability for the old counting rules. This was calculated by the intraclass correlation coefficients (ICC) between the averaged counting of observer 1 (KM) and 2 (VC).

Cancer	Parameter	Samples	ICC
**CRC**	V	285	0.76 (0.65–0.83)
**CRC**	N	285	0.54 (0.45–0.63)
**CRC**	Q_A_	19	0.24 (0.09–0.40)
**CRC**	A_A_	19	0.38 (0.05–0.63)
**GBM**	V	300	0.86 (0.83–0.89)
**GBM**	N	300	0.73 (0.67–0.78)
**GBM**	Q_A_	20	0.60 (0.35–0.75)
**GBM**	A_A_	20	0.80 (0.56–0.85)
**OC**	V	315	0.85 (0.80–0.89)
**OC**	N	315	0.70 (0.62–0.76)
**OC**	Q_A_	21	0.51 (0.33–0.63)
**OC**	A_A_	21	0.74 (0.65–0.86)
**RCC**	V	330	0.64 (0.58–0.71)
**RCC**	N	330	0.73 (0.69–0.77)
**RCC**	Q_A_	22	0.70 (0.57–0.80)
**RCC**	A_A_	22	0.58 (0.44–0.70)

CRC, colorectal cancer; GBM, glioblastoma multiforme; OC, ovarian cancer; RCC, renal cell cancer; V, the number of grid points overlapping with vessels in one region of interest (ROI); N, the number of microvessel profiles in one ROI; Q_A_, the microvessel density based on N in one sample (15 ROIs); A_A_, the microvessel areal fraction based on V in one sample (15 ROIs); (LoCI—UpCI), the 95% lower and upper confidence intervals

**Table 4:** Inter-observer variability for the new counting rules. This was calculated by the intraclass correlation coefficients (ICC) between the counting of observer 1 (KM), 4 (PV), 5 (YW) and 6 (WW).

Cancer	Parameter	Samples	ICC1 (1–4)	ICC2 (1–5)	ICC3 (4–5)		ICC4 (1–6)	ICC5 (5–6)	ICC6 (4–6)
**CRC**	N	270	0.90	0.85		0.74	NA	NA	NA
**CRC**	V	270	0.85	0.80		0.72	NA	NA	NA
**CRC**	Q_A_	18	0.91	0.82		0.70	NA	NA	NA
**CRC**	A_A_	18	0.90	0.77		0.72	NA	NA	NA
**OC**	N	300	0.92	0.71		0.79	0.73	0.81	0.83
**OC**	V	300	0.84	0.88		0.84	0.83	0.87	0.78
**OC**	Q_A_	20	0.95	0.72		0.81	0.77	0.89	0.89
**OC**	A_A_	20	0.94	0.97		0.95	0.89	0.93	0.84

ICC, intraclass correlation coefficient; CRC, colorectal cancer; OC, ovarian cancer; N, the number of microvessel profiles in one region of interest (ROI); V, the number of grid points overlapping with vessels in one ROI; Q_A_, the microvessel density based on N in one sample (15 ROIs); A_A_, the microvessel areal fraction based on V in one sample (15 ROIs); NA, not available

## Discussion

Manual ROI sampling and blood vessel counting is a time consuming and labor intensive process, partly due to the amount of effort required to find valid ROIs under the microscope. Although there are different methods for sampling and counting, the validation is usually limited to one specific cancer type. It is important to note that the sampling method chosen depends on the research question, as the hotspot method (Weidner’s method) will quantify the strongest angiogenic areas of a tumor and the SURS method provides global information. We developed a novel ROI sampling and microvessel counting method that combines parts from existing methods and adds stereological techniques to improve the validity of the results. Furthermore, we used WSIs of CD31-stained tissue sections, allowing traceability and higher throughput by providing ROI annotations on the images [19]. The level of consensus within and between observers was evaluated by calculating the ICCs, which were higher than the generally accepted minimal reliability of 0.7 [27] and more than half of the results even exceeded 0.8. These results were only possible after consensus training with all observers and with the new counting rules. Therefore, we strongly advise simplified counting rules and extensive consensus training sessions with all observers involved. Most attention needs to be paid to the minimum size of a staining pattern that can be considered a vessel. Our newly proposed counting rules include every CD31-positive object, no matter how small, and therefore also has the advantage of including single endothelial venules, indicative of active angiogenesis [28].

We evaluated the effect of the location of ROIs on the variability of the microvessel density and areal fraction of the blood vessels. No major effect was present if SURS is performed for profile counting. However, for the areal fraction, ICC-values lower than 0.7 were obtained. This is not unexpected, as the amount of overlapping structures with the grid will depend heavily on these locations as only a small area is sampled by the grid intersections. In addition, these ICCs can be regarded as too optimistic because only one observer assessed the ROIs. Therefore, additional intra-observer variability could be taken into account. We limited our investigation to the above-mentioned parameters, but it would be interesting to study the effect of magnification, grid type or grid size in future research.

A limitation to our study is the relatively small sample size and therefore a follow-up study with more samples is advised.

Several challenges are inherent to vessel counting independently of the method used: firstly, tumors develop in different tissue types, which all have their own characteristic vessel network architecture [29–32]. The distribution of microvessel sizes and growth patterns vary between, but also within, a cancer type [29,32,33]. Starting from the first published milestone study by Weidner N et al. [15] many published research studies have been conducted regarding the significance of microvessel density in breast cancer patients. It is of great interest to evaluate our method in a follow-up study with breast cancer samples. Secondly, the different cell types of which microvessels are composed are another source of bias [34–38]. The proportions of the different cell types in a vessel define the vessel type. The most important cell type is the endothelial cell [39]. Peri-endothelial cells, such as pericytes and smooth muscle cells, strengthen the vessel and expand its functionalities [34,40]. The importance of these different types of vessels present in the growing tumor tissue seems to be prognostic [41,42] and even predictive of survival after therapy [24,43–45]. Developing an assay for the detection of these pericytes in blood vessels would be of great interest, but is challenging. For example, alpha-smooth muscle actin, which stains pericytes, also stains myofibroblasts hindering quantification. Future studies will be needed to address the challenges imposed by such a strategy. Because the choice of vascular cell type that is stained can lead to the selection of a specific type of vessel in terms of functionality, this choice will also affect the number of the microvessels counted. Most microvessels are stained in the tumor sections using pan-endothelial markers such as CD31. However, these proteins are not only expressed by endothelial cells, but also by other cells, such as macrophages and platelets [12], which may result in an overestimation of the number of microvessels. Thirdly, the counting method has sources of bias as well, because a decision has to be made whether stained cells resemble endothelial cells in shape and size. Depending on the orientation of the vessel and the direction of the sectioning, one vessel can appear as separate shapes in the two-dimensional section. Finally, there are several challenges presented by manual counting per se, such as searching and finding microvessels, counting and memorizing the number of counts [46,47].

Nonetheless, the present investigation shows that it is possible to obtain an unbiased result by our method. Moreover, the validity of the method described in the present study was shown in the GOG-0218 trial [48], in which patients with epithelial ovarian cancer were treated with carboplatin-paclitaxel with or without bevacizumab (manuscript in preparation). Higher microvessel density values in the CD31-stained samples that were measured by our method showed prognostic and potential predictive value for progression-free survival [48]. In conclusion, the present microvessel counting method is reliable if observers are extensively trained. Although the amount of ROIs needed depends on the cancer type, on average ten ROIs are sufficient for accurate microvessel density measurements.

## Supporting Information

S1 Dataset Counting results. The results for the four parameters (N, MVD, V,A_A_) for the samples from the four cancer types (CRC, GBM, OC, RCC) from all observers (1–6) obtained with both versions of the counting rules are contained in these CSV-files.
(ZIP)
